# Increasing the Sensitivity of pH Glass Electrodes with Constant Potential Coulometry at Zero Current

**DOI:** 10.1021/acs.analchem.4c00592

**Published:** 2024-04-09

**Authors:** Robin Nussbaum, Stéphane Jeanneret, Eric Bakker

**Affiliations:** Department of Inorganic and Analytical Chemistry, University of Geneva, CH-1211 Geneva, Switzerland

## Abstract

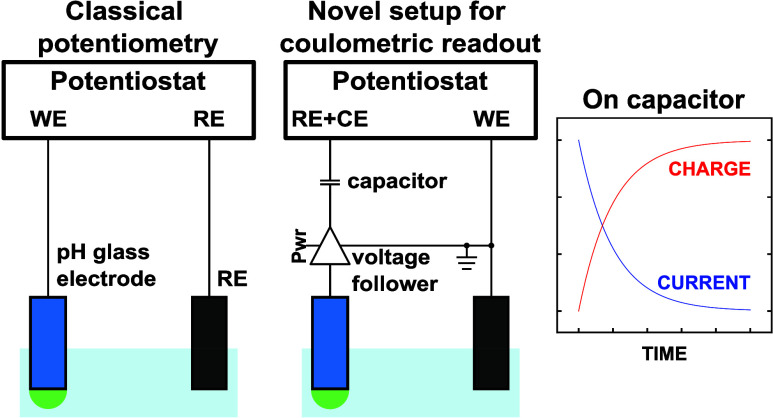

It has recently become possible to increase the sensitivity of ion-selective electrodes (ISEs) by imposing a constant cell potential, allowing one to record current spikes with a capacitor placed in series in the circuit. The approach requires a transient current to pass through the measurement cell, which unfortunately may introduce measurement errors and additionally excludes the use of high-impedance indicator electrodes, such as pH glass electrodes. We present here an electronic circuit that overcomes these limitations, where the cell is measured at zero current in combination with a voltage follower, and the current spike and capacitor charging occur entirely within the instrument. The approach avoids the need for a counter electrode, and one may use any electrode useful in potentiometry regardless of its impedance. The characteristics of the circuit were found to approach ideality when evaluated with either an external potential source or an Ag/AgCl electrode. The current may be linearized and extrapolated to further reduce the measurement time. The circuit is further tested with the most common yet very challenging electrode, the pH glass electrode. A precision of 64 μpH was obtained for 0.01 pH change up to 0.05 from a reference solution. Similar pH changes were also measured reliably further away from the reference solution (0.5–0.55) and resulted in a precision of 377 μpH. The limitations of this experimental setup were explored by performing pH calibrations within the measuring range of the probe.

Ion-selective electrodes (ISEs) are established analytical tools for ion sensing in complex samples.^1,2^ They are usually operated at zero current, allowing one in ideal cases to correlate the potential at the electrode–solution interface to the ion activity in the sample solution. However, the sensitivity of ISEs is dictated by the Nernst equation and corresponds to 59.2 mV for positive singly charged ions at 25 °C. This limited sensitivity can be challenging in some applications. For example, sodium concentration in blood ranges from 135 to 145 mmol/L.^3^ A sodium level outside of this range can be harmful, and it is therefore important to monitor accurately the concentration within its narrow range.^4,5^ Another important example is the measurement of the pH in oceans. Increased carbon dioxide input into the atmosphere from anthropogenic sources results in surface oceanic pH values that slowly decrease over time at a rate of about −0.002 pH per year.^6,7^ This has an impact on metal speciation and calcification processes.^8,9^ It is therefore crucial to monitor these small pH changes at an adequate resolution. Today, however, such small changes are challenging to measure reliably.

The limited sensitivity of ISEs can be overcome with alternative readouts mostly using dynamic electrochemistry techniques.^10^ An improved sensitivity for pH glass electrodes was reported using a 4-electrode setup under nonequilibrium conditions.^11^ The approach offered both potentiometric and amperometric detection modes. While the potentiometric slope was increased by a factor of 10, the addition of an extra electrode compared to the classical 3-electrode setup remains an important drawback. An enhanced potentiometric response for chloride, fluoride, and pH electrodes was also achieved using an expanded electronic circuit,^12^ giving an increased sensitivity by almost a factor of 100. However, this improvement required multiple ISEs (30 for chloride and 10 for both other ions) to be connected. More recently, amperometry with an inverted electrode configuration was successfully applied to increase the sensitivity of ISEs.^13^ The ISE was used as a reference electrode (RE), while an Ag/AgCl element was treated as a nonpolarizable working electrode (WE). This method gave a linear dependence on concentration within a limited concentration range and high-resolution pH sensing was also achieved.^14^ The linear dependence of the signal on concentration may be perceived as a drawback because the pH scale is, by definition, logarithmic.

Increased sensitivity may also be obtained by constant potential coulometry, which was first introduced for solid-contact ISEs in 2015 using a classic three-electrode setup.^15^ It originally took advantage of the capacitive properties of the ion-to-electron transducing layer to obtain an amplified signal compared to classical zero current potentiometry.^16^ As the name implies, a constant potential is imposed between the ISE and the reference electrode. Thus, any phase boundary potential change at the ISE results in an opposite potential change on the capacitive transducing layer and gives rise to a transient current. The charge of the current spike is then used as an analytical signal and can be predicted using eq 1(17)
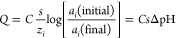
1where *Q* is the charge from the integrated transient current, *C* is the capacitance of the capacitive element, *s* is the Nernstian slope for a monovalent ion, *z*_*i*_ is the charge, and *a*_*i*_ is the activity of the ion *i*. This approach was further developed by implementing an electronic capacitor in series with the ISE, removing the need to use solid contact ISEs and allowing for electronic control of the capacitive element (Scheme 1a).^18,19^ However, the requirement of flowing current through the working electrode made it impossible to use pH glass electrodes because of their high impedance. While polymeric membrane-based ISEs exhibit lower impedance, it was recently reported that current flow through such membranes results in membrane polarization and an undesired change in the phase-boundary potential.^20^ More recently, inverted configurations for constant potential coulometry were reported using a dummy ISE or an Ag/AgCl element as WE, avoiding current flow through the ISE.^21,22^ Unfortunately, however, the reference input of a potentiostat is not designed for a highly resistive element, such as a pH glass electrode.

**Scheme 1:**
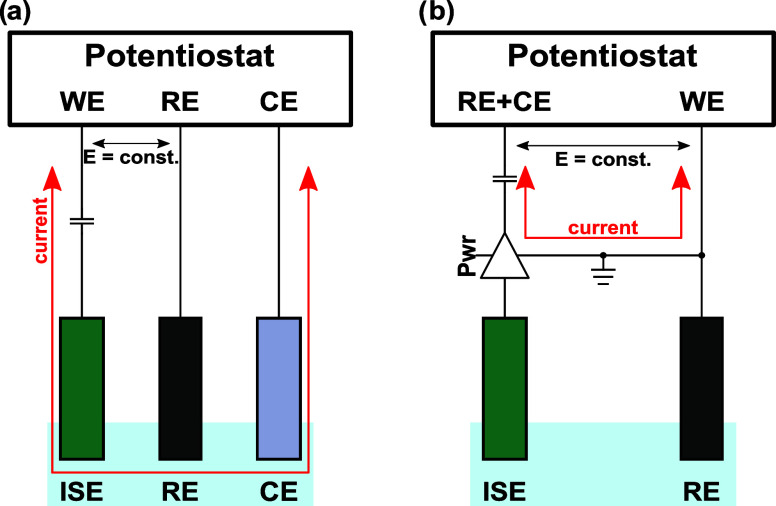
Experimental Setup Used in (a) Previous and (b) Current Work The present approach avoids the passage of current through the measurement cell by using a voltage follower.

In this work, we propose a novel electronic circuit for constant potential coulometry that separates the capacitive current entirely from the electrochemical cell (Scheme 1b). The key element is a voltage follower on the ISE connector, allowing for the first time the isolation and use of high input impedance electrodes, such as pH glass electrodes. This now avoids any influence of the current on the indicator electrode and gives an amplified signal. The behavior of the circuit is investigated. The sensitivity of the proposed setup is evaluated for chloride sensing as a model system and compared with zero current potentiometry. As proof of concept, the system is then successfully applied to pH sensing in different ranges using a glass electrode.

## Experimental Section

### Materials and Instrumentation

Citric acid monohydrate and potassium nitrate (KNO_3_) were purchased from Arcos Organics. Boric acid, disodium phosphate (Na_2_HPO_4_), lithium acetate (LiOAc), and sodium chloride (NaCl) were purchased from Sigma-Aldrich (Merck). Hydrochloric acid conc. 37% (HCl) was purchased from Fisher Chemical. Volumetric 1 M sodium hydroxide (NaOH) solution was purchased from Thermo Scientific. 3 M potassium chloride (KCl) solution was purchased from Metrohm.

The pH glass electrodes were generously supplied by Metroglas AG, Switzerland. They are made of very robust glass with a long lifetime, similar to the ones sold to Idronaut, Italy, for implementation in submersible probes. Their impedance was determined to be around 50 MΩ. Silver chloride electrodes were prepared as previously reported.^22^ Potentiometry experiments were performed with a high-impedance input 16-channel EMF monitor (Lawson Laboratories, Inc., Malvern, PA) with a double junction Ag/AgCl/3 M KCl/1 M LiOAc reference electrode (Metrohm, Switzerland). Chronoamperometry with an electronic capacitor was performed with an Autolab PGSTAT302N (Metrohm Autolab) and a homemade electronic circuit placed between the cell and the instrument. The current was sampled over a period of at least 5 times the RC (resistance multiplied by capacitance) time constant to ensure complete charging of the capacitor. The circuit is described in more detail below. The open circuit potential (OCP) could also be recorded with the Autolab during the calibrations. When the performance of the circuit was tested, the pH changes were simulated with a 2450 Source Measure Unit (SMU) instrument (Keithley, Tektronix), which was connected to the electrode inputs of the circuit. Automated pH titrations of buffers were performed with a 765 Dosimat (Metrohm, Switzerland) controlled with custom LabView software. Automated additions during the calibrations were done with an 800 Dosino (Metrohm, Switzerland).

### Calibrations and Titrations

All calibrations and titrations were done in a closed and temperature-controlled glass cell at 25 °C. The starting volume was always 30 mL. The NaOH solutions were all diluted from standardized 1 M NaOH. The chloride calibration was performed in 100 mM NaCl with 100 mM KNO_3_ as background and bridge electrolyte in the reference electrode. The chloride concentration was altered by the addition of 1 M NaCl. The volume change was less than 2%. The pH calibration over a wide range was done in a 40 mM universal buffer solution (UBS) with a starting pH of 3.71 and ended at 10.07. The required volumes to reach a given pH value were determined by automated titration of said buffer with 1 M NaOH. The pH calibrations with 0.01 pH steps (0–0.05 and 0.5–0.55) were performed in 20 mM boric acid buffer containing 10 mM NaCl at pH = 8.21. Its p*K*_a_ was determined experimentally by automated titration with 100 mM NaOH and was equal to 9.16. The pH was then precisely adjusted to p*K*_a_-0.95 (8.21) with 20 mM NaOH solution. The required addition volumes of 10 mM NaOH solution for 0.05 ΔpH calibrations were determined with the Henderson–Hasselbalch equation and resulted in a volume change of less than 5%.

### Electronic Circuit

A novel homemade electronic circuit, named *CapaBoard*, was designed to be placed between the electrochemical cell and the potentiostat. Detailed schemes with all of the connectors, relays, and pictures can be found in Figures S1, S2, S3, and S4. The circuit was connected as described in Figure S1. The dimensions of the box are 19.6 cm × 10.7 cm × 3.6 cm with a mass of 435 g. It contains four resistances (1, 2, 5, 10 kΩ), four polarized tantalum capacitors (48.8, 97.8, 193, 482 μF), a direct input, two voltage followers of different sensitivity (only the first one was used in this work), and various relays controlled by a microcontroller. The transient currents were such that the capacitors were not damaged during the measurements. The box was powered and connected to the computer via USB and controlled by using Nova 2.1.4 software (Metrohm Autolab).

**Scheme 2:**
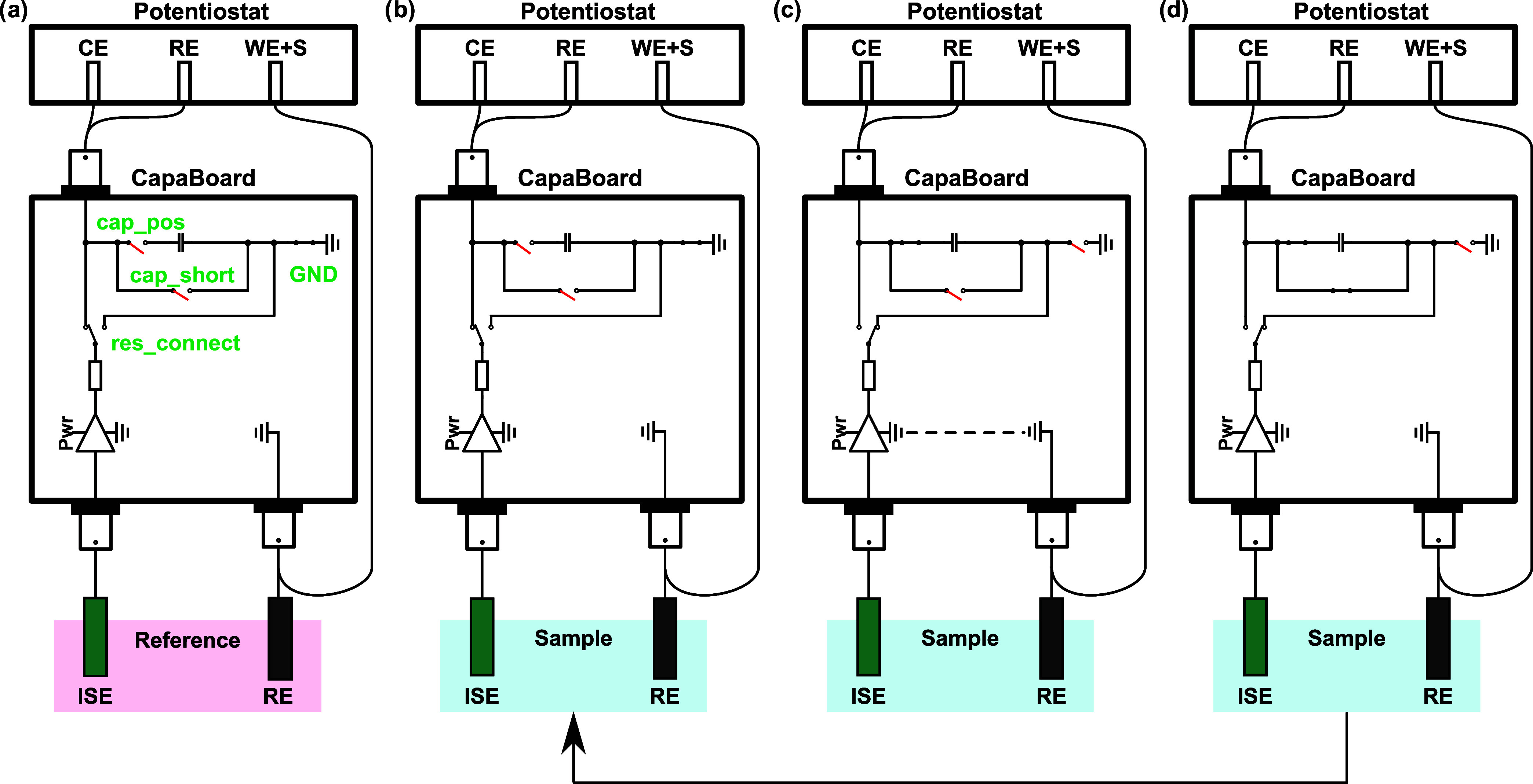
Electronic Procedure Used during Coulometric Calibrations (a) Measurement of reference OCP, (b) measurement of sample OCP, (c) enforcing of OCP_ref_ and transient current measurement, (d) discharge of the capacitor.

During coulometric calibrations, the following sequential steps were executed (see Scheme 2). The OCP in a reference solution was first determined and stored in the potentiostat (Scheme 2a). An addition was then performed to change the analyte activity, inducing a phase boundary potential change at the ISE. The OCP was again measured in this sample solution (Scheme 2b). To perform the coulometric measurement, the *res_connect* relay was changed to the right position, the ground (GND) relay was disconnected, the OCP_ref_ was enforced by the potentiostat and the *cap_pos* relay was connected to measure the charging current on the capacitor (Scheme 2c). The *res_connect* is the most crucial switch in the circuit, as it allows one to impose the OCP_ref_ on one side of the capacitor and the sample OCP on the other side. Thus, the capacitive current results from the potential mismatch in the circuit, as it has been the case with three-electrode constant potential coulometry. Finally, the potential enforcement was stopped, and the *cap_short* relay was connected to allow the short circuiting of the electronic capacitor (Scheme 2d). One may then return to step (b) to perform replicates in the same sample or change the solution to continue with a new measurement. The OCP_ref_ was also enforced in the reference solution to determine and subtract the residual charge from the instrument. It is important to note that step (b) is optional and is only used to compare potentiometry to coulometry in this study. If the potential stability of the probe is adequate, one may directly loop back from step (d) to (c).

## Results and Discussion

**Figure 1:**
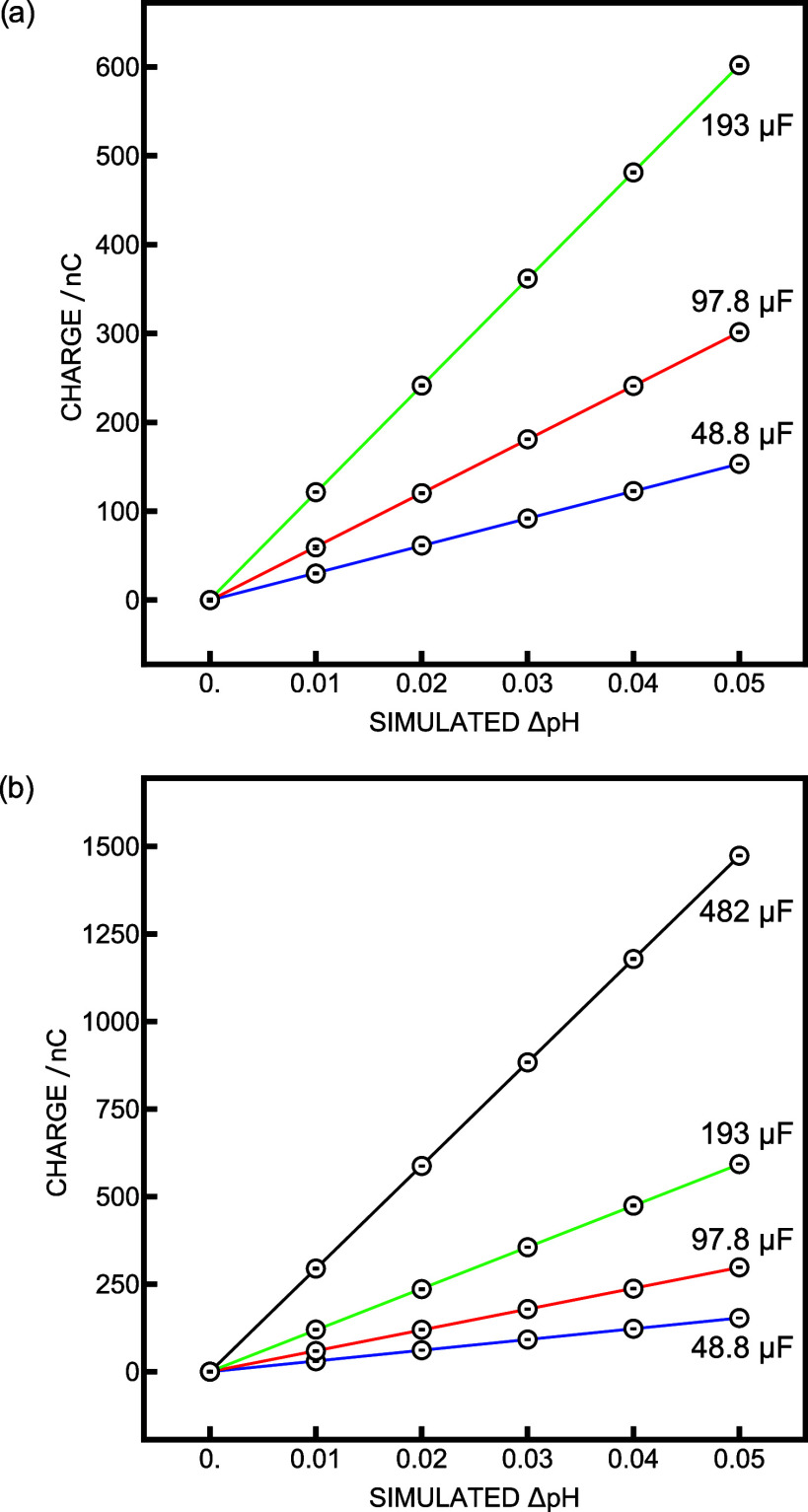
Calibration curves obtained with the source meter as potential input while (a) changing the capacitance (C) and keeping the resistance (*R*) constant at 10 kΩ and (b) changing both *C* and *R* to keep the *RC* time constant around 0.5 s.

Previous work on constant potential coulometry with ISEs all used a three-electrode setup, with current flowing either through the sensing electrode or a dummy electrode (Scheme 1a).^15−22^ This typical electrode configuration was required to avoid current flow through the reference electrode to keep its potential untouched. In this work, we propose a novel electronic circuit that uses a two-electrode setup (Scheme 1b). In fact, the voltage follower ensures that no current flows through the measurement cell, eliminating the need for a counter electrode. The potential difference between the electrodes alters the capacitive current without a current flow through the cell. The behavior of the electronic circuit was first investigated using an SMU instrument to impose potentials on the connectors where the two electrodes should be, as this allowed for a repeatable and stable potential input. The potential was varied to mimic pH changes of 0.01 (592 μV steps) up to 0.05. The capacitance was modified with an added constant resistance value of 10 kΩ (Figure 1a). Increasing the capacitance resulted in longer measurement times because it increased the RC time constant, which was not convenient to perform multiple replicates rapidly. Therefore, the resistance was modified accordingly to maintain an RC time constant of about 0.5 s (Figure 1b).

The obtained precision data for different configurations are given in Table 1. The charges agree well with theory (eq 1, deviation from the slope <7%), and the precision increased with increasing capacitance. This initial study allowed us to confirm the advantage of avoiding current flow through the electrodes. In fact, it was recently demonstrated that flowing current through membrane electrodes induced the diffusion of ions and altered the phase boundary potential, which gives rise to measurement errors.^20^ The higher the current, the more important is the ion flux. Even when an Ag/AgCl electrode is used in an inverted configuration, an excessive current may impact the AgCl layer and modify its properties. In these works, it was therefore important to attenuate the current to minimize the potential drift, which was achieved by adding a resistance in series after the WE. As the charging time depends on both the resistance and the capacitance, the latter was kept relatively small to avoid excessive measurement times. Here, on the other hand, while the capacitive current is still dictated by the potential between the electrodes, the measurement cell is no longer subject to current flow. For this reason, there is no current-induced potential change at the ISE and the only limitations are the characteristics of the electronic components. This allows one to tolerate much larger currents and use larger capacitances, resulting in an increased analytical signal.

**Table 1:** Precision Obtained with the SMU Instrument for Different Resistance and Capacitance Combinations

resistance/kΩ	capacitance/μF	RC time constant/s	precision/μpH
10	193	1.9	93
10	97.8	1.0	132
10	48.8	0.5	210
5	97.8	0.5	200
2	193	0.4	155
1	482	0.5	53

Previous work from our group on constant potential coulometry focused on chloride sensing using a silver/silver chloride electrode and on sodium and pH sensing using membrane-based electrodes as WE.^18−20^ Our latest work focused on using the ISE as the RE and an Ag/AgCl element as WE because it is largely insensitive to polarization.^22^ This setup was demonstrated to improve the reproducibility of the measurements but required one to maintain a constant background chloride activity in the sample, which is inconvenient. Moreover, the resistance of the RE was found to influence the noise of the current signal. The high input impedance of typical pH glass electrodes (tens of MΩ) makes it equally impossible to connect the indicator electrode to the reference electrode input of the potentiostat. Expanding the attractive constant potential coulometric method to glass pH electrodes to improve their sensitivity would seem very important because it is the most established class of ISEs in use for over a century and the *de facto* reference standard for pH measurements.^23,24^ The above-mentioned limitations should be overcome with the electronic circuit proposed here, thanks to the high-impedance input of the voltage follower. In principle, therefore, any ISE may now be interrogated by the constant potential coulometric method.

**Figure 2:**
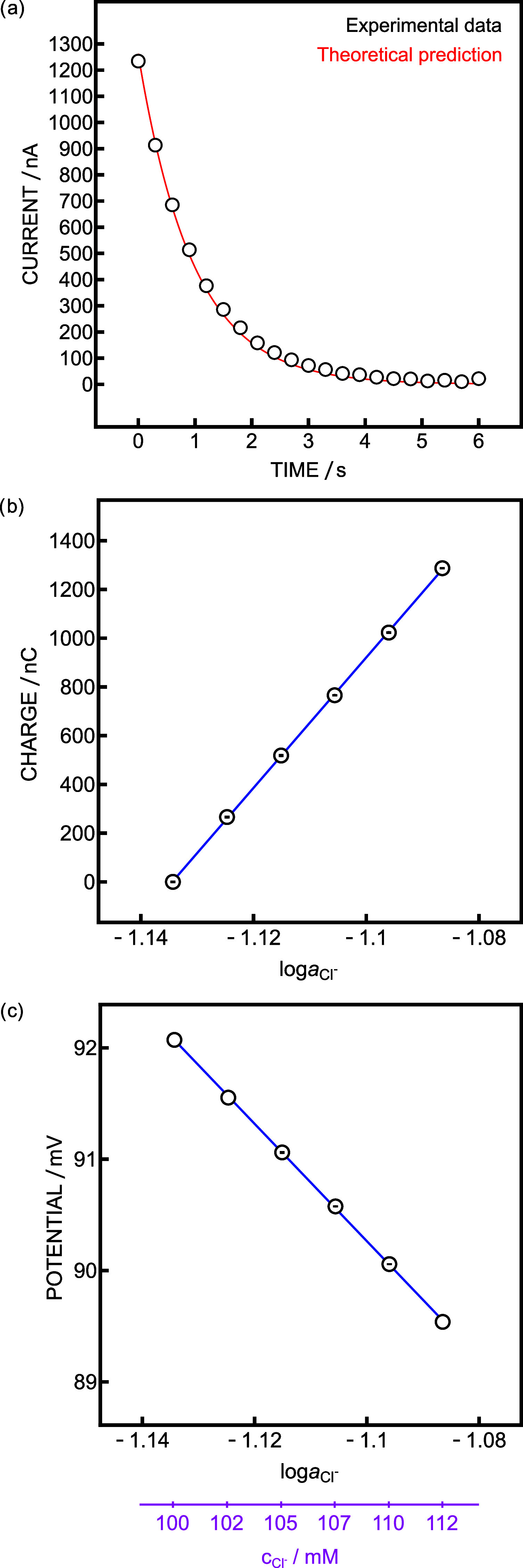
(a) Transient current observed at 112 mM Cl^–^ (dots) and theoretical fit (red). Chloride calibration curves obtained with (b) coulometry with *C* = 482 μF and *R* = 2 kΩ and (c) zero current potentiometry (*N* = 5). The absence of error bars in panel (c) reflects the resolution limit of the potentiometer (see text).

First, the circuit was used to measure chloride with a silver chloride electrode (see the Experimental Section for the preparation procedure) and a double junction reference electrode. As the resistance of the double junction was much higher than that of the Ag/AgCl element, it was the latter electrode that was connected to the ground input of the CapaBoard. This configuration resulted in significantly reduced current noise, establishing the best conditions for comparison with zero current potentiometry and current fitting. The response of the electrode was evaluated with constant potential coulometry with a capacitance of 482 μF and a resistance of 2 kΩ in the concentration range from 100 to 112 mM NaCl in steps of 0.01 log* a*_Cl_^–^. This concentration range was chosen as it gave stable OCP readings in previous work.^22^ The current was recorded for a time period of 6 RC to ensure complete charging of the capacitor. The current spike observed for 112 mM is shown in Figure 2a. The experimental current fit to an ideal RC decay curve was excellent, confirming the suitability of the electronic circuit for measurements with ISEs. For each concentration, 5 coulometric pulses and OCP measurements were performed (Figure 2b,c). The obtained slopes were 26,800 nC (ideal theory: 28,800 nC) and −52.7 (−59.2 from theory). The obtained precisions were 71 and 38 μlog* a*_Cl_^–^. At first sight, zero current potentiometry appears to exhibit excellent reproducibility, but this is misleading as the apparent increased precision results from the limited resolution of the potentiometer. One may notice in Figure 2c that some points lack error bars. This indicates that the potential variation for 5 replicates for a given chloride concentration was smaller than the instrument resolution. The lowest potential step that could be monitored was 30 μV, corresponding to an activity change of 500 μlog* a*_Cl_^–^, which is about 7 times larger than the precision obtained from coulometry.

**Figure 3:**
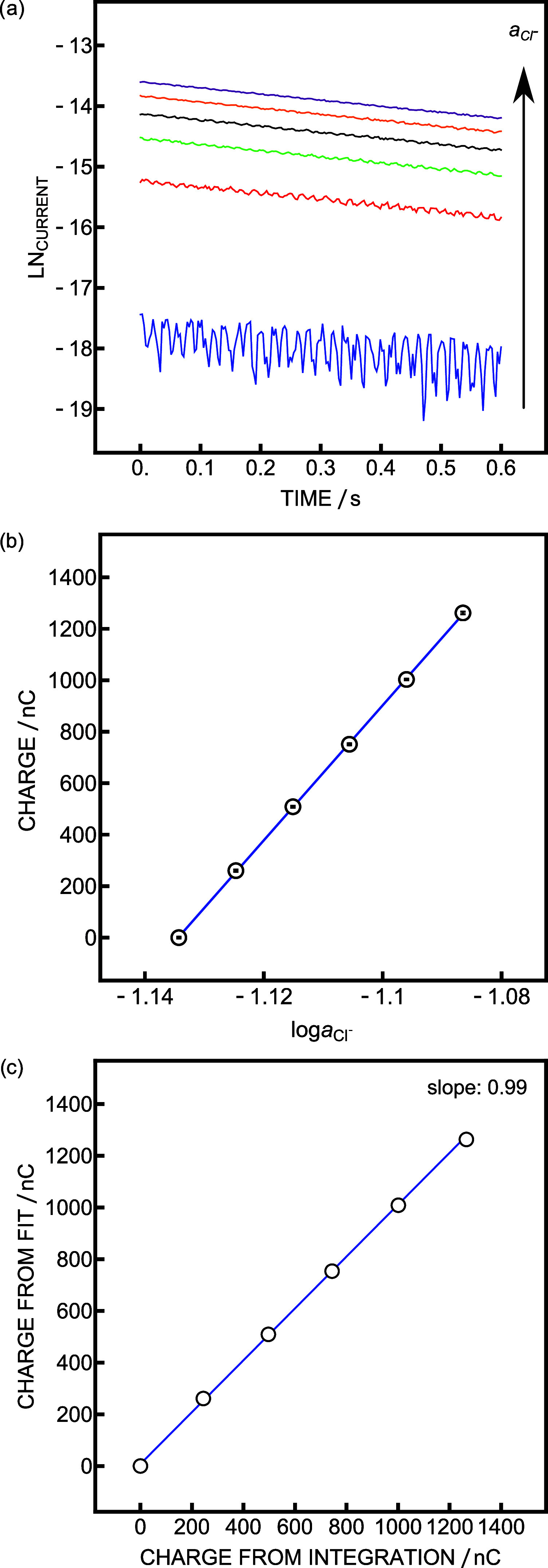
(a) Linearized current trace up to 0.6 RC obtained during chloride calibration with (b) the corresponding charge plot obtained from the extrapolation at 6 RC. The linearization was achieved by taking the natural logarithm of the current. (c) Correlation between charge obtained from integration (*x-*axis) and the one obtained from current linearization (*y-*axis).

Current linearization was previously demonstrated when the capacitive current fit was excellent.^19^ This was achieved by taking the natural logarithm of the current and extrapolating to long times, allowing one to reduce the measurement time, as a complete charging of the capacitor was not required. This approach was explored here with the chloride calibration data from Figure 2b. The current was sampled and linearized over a short period (*t* = 0.6 RC) and extrapolated to *t* = 6 RC (Figure 3a). As the peak current increases with chloride activity, the noise becomes less apparent with increasing chloride concentration. The charge was then obtained according to the previous work (Figure 3b).^19^ The correlation between experimental charge and fitted charges was excellent (slope of 0.99), but it did not result in an improved precision, with values of 71 and 149 μlog *a*_Cl_^–^ respectively (Figure 3c). For this reason, the approach was not applied to further data analysis, and the integral over the full time period was taken as an analytical signal to evaluate measurement reproducibility. The results confirm, however, that the sampling time may be significantly reduced to allow for faster sampling at the cost of decreased precision.

**Figure 4:**
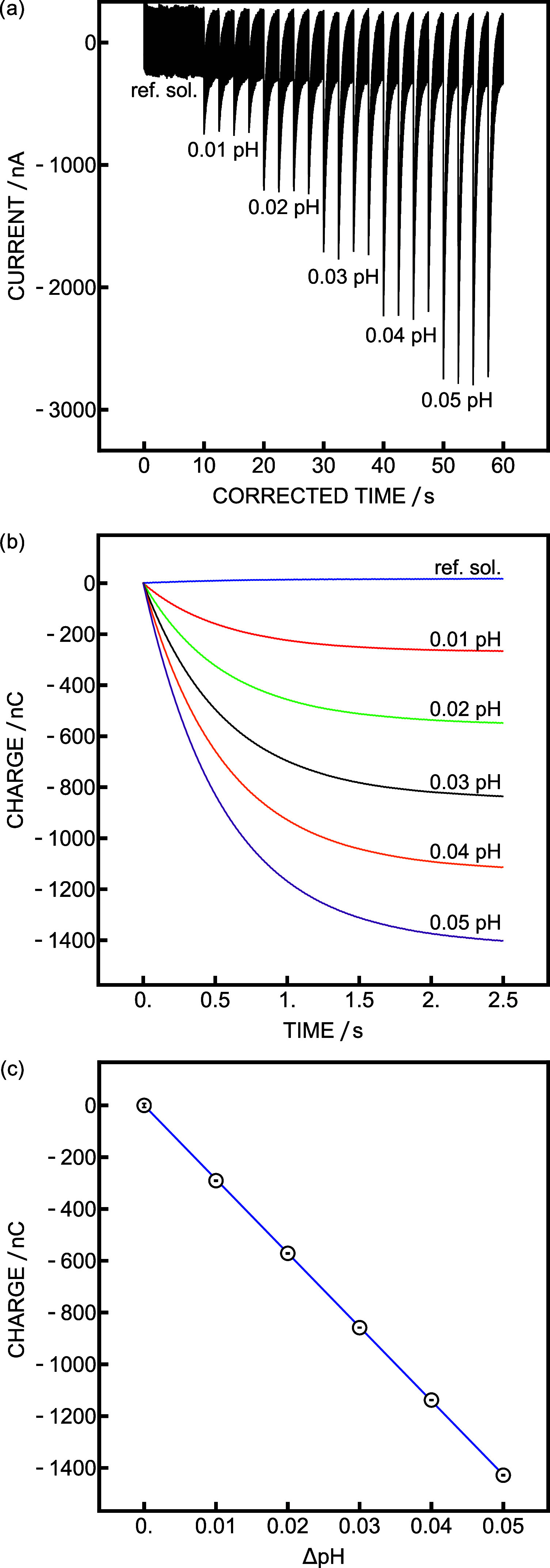
(a) Current trace of coulometric calibration with *C* = 482 μF and *R* = 1 kΩ. (b) Charge obtained during the coulometric calibration at different pH values. (c) Coulometric calibration curve.

As discussed above, pH glass electrodes are the most commonly used electrodes and their impedance is among the highest.^24^ It was therefore an application of choice to demonstrate the capability of the CapaBoard. As this approach aims for in situ measurements, glass electrodes of the exact formulation used in commercial aquatic submersible probes were sourced from Metroglas AG, Switzerland (see the Experimental Section). The response of these electrodes was first evaluated by zero current potentiometry over a wide pH range (3.71–10.07) in 40 mM UBS (Figure S5a) and in a boric acid buffer over a more limited 0.05 pH range (Figure S5b). The obtained slopes were, respectively, 56.5 and 59.5 mV, which agrees well with the Nernst equation. The required volumes for both calibration ranges were determined, as discussed in the Experimental Section. The automated pH titrations are shown in Figure S6. Excellent precision was already reported for 1 mpH steps away from the reference solution, but this is not how glass electrodes tend to be calibrated.^18^ Therefore, this work aims to demonstrate excellent precision with larger pH changes. A coulometric pH calibration with increments of 0.01 pH units, a 482 μF capacitor, and a 1 kΩ resistance was performed for a pH change up to +0.05 pH. The current traces are presented in Figure 4a. The noise was higher than that in previous work, but because the integrated current was used as the analytical signal, the noise had minimal impact on precision. This noise increase was due to the increased resistance on the reference electrode connected to the GND of the CapaBoard. Using a simple Ag/AgCl element as the reference electrode in combination with the pH glass electrode did indeed reduce the noise but did not result in better precision. Individual time-dependent charges are listed in Figure 4b. The respective calibration curve is shown in Figure 4c, giving a precision of 64 μpH, which is significantly better than that reported in previous work (177 μpH^22^). This improvement is a direct consequence of separating the potential measurement from the capacitive current because one may now use a bigger capacitor (482 μF) compared to the previous study (100 μF^22^), as emphasized previously. A similar calibration was carried out by connecting the pH glass electrode to the direct channel (Figure S1) to explore the behavior of the circuit with the original protocol but without a voltage follower. No appropriate capacitive current was seen during the calibration procedure (Figure S7), demonstrating that a voltage follower is needed to overcome the high impedance of the glass electrode.

**Figure 5:**
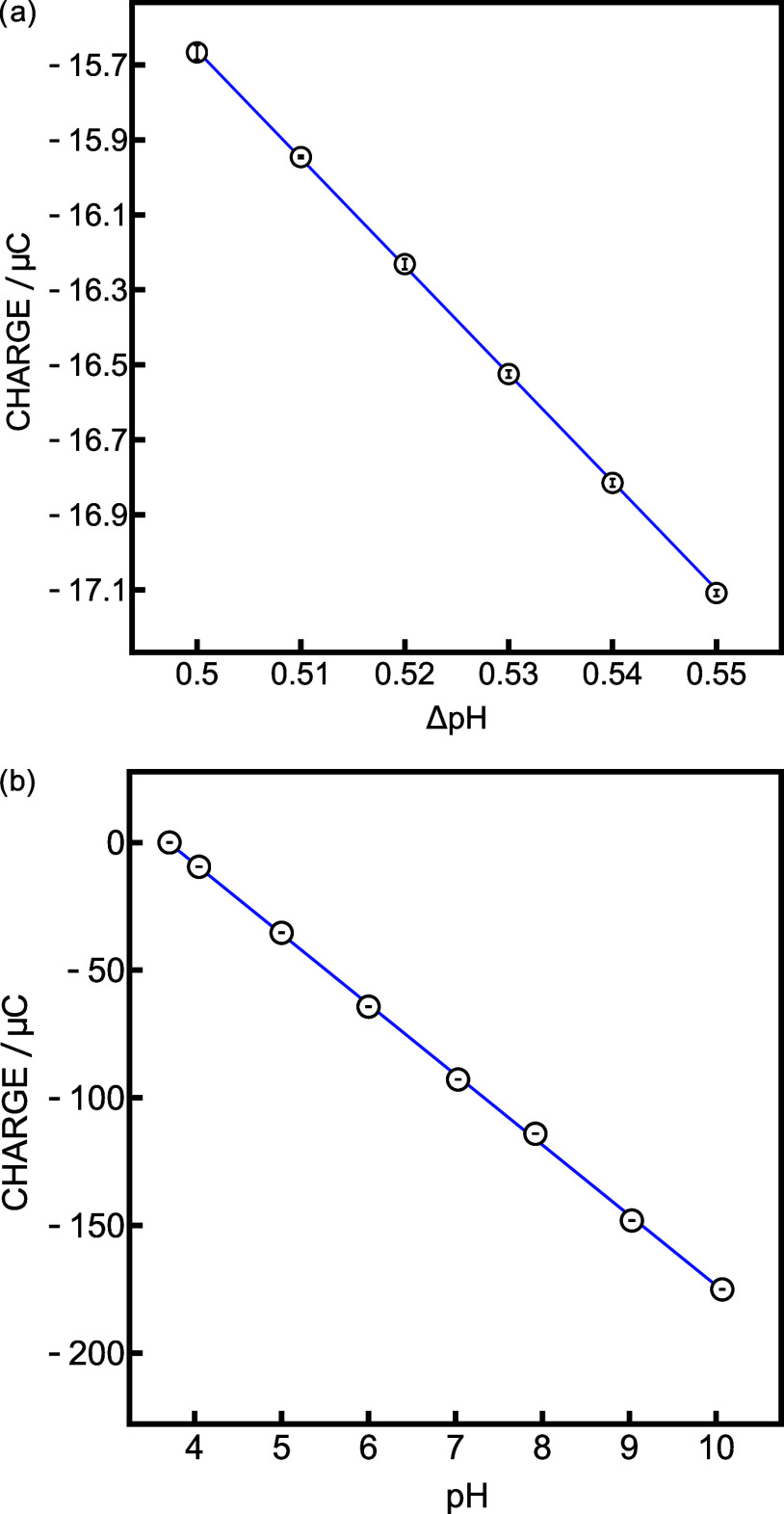
Coulometric calibration curves with *C* = 482 μF and *R* = 1 kΩ (a) between 0.5 and 0.55 ΔpH and (b) over a wide pH range.

The ability to tolerate higher currents with this new setup should also allow for larger activity changes that would otherwise induce excessive current amplitudes. Not being limited to very small activity changes offers a significant advantage compared to previous work because, in practice, the calibration solution pH can rarely be maintained very close to that of the sample. This is especially important when aiming for precise in situ pH measurement because external factors can vary rapidly and influence the measurement. In pH determination, the temperature influences the p*K*_a_ of the reference buffer and the sample pH. As the current in constant potential coulometry is directly dependent on the potential difference between the reference and sample solution, the coulometric signal is thus expected to change with the temperature. The measurement system should be able to handle these current fluctuations. To assess this aspect, a calibration further away from the reference potential (0.5–0.55 ΔpH) was performed in the same boric acid buffer (Figure 5a). The obtained precision (377 μpH) decreased compared with the measurement closer to the calibration point. This can be explained by the increased charges, which result in a higher average standard deviation. However, it was still lower than the resolution of classical potentiometry (500 μpH). This demonstrated the robustness of the method for measurements in a less controlled environment and is promising for everyday applications. The limits of this new setup were further evaluated for pH calibration in a very broad range (3.71–10.07). The reference solution was chosen to be equal to the initial sample composition of the calibration. The resulting coulometric calibration is presented in Figure 5b. The successful results demonstrate the universality of the presented system and its ability to accommodate high current flow as high as hundreds of μA. Here, however, the precision was reduced to 940 μpH. As the charges become much larger, they cannot be compared with the excellent precision obtained with smaller calibration ranges.

Constant potential coulometry was initially developed to improve the sensitivity of ISEs whose sensitivity is limited by the Nernstian response slope and the best results are indeed obtained with small activity changes.^16,18^

## Conclusions

A novel circuit for constant potential coulometry was developed, characterized with an SMU instrument, and applied to chloride and pH sensing with improved precision compared to previous work. The theoretical RC fit of the current was excellent, and the charge could also be predicted by current linearization, reducing the measurement time at the expense of decreasing precision. The high-impedance channel of the voltage follower allows one to perform constant potential coulometry with a routine pH glass electrode. The ability to record large current spikes was assessed with a calibration farther away from the reference solution (0.5–0.55) and resulted in an attractive precision. On a much bigger pH range (3.97–10.07), the method could still be used successfully, which is a first for constant potential coulometry, but the precision became progressively worse with the calibration pH value departing from that of the sample solution pH. The universality and versatility of the newly proposed setup may improve the sensitivity of many ISEs and remove the requirement of a counter electrode for constant potential coulometry. We note that any systematic errors cannot be compensated by this approach, which may include limited membrane selectivity, errors at the liquid junction of the reference electrode, signal drift, and temperature fluctuations. The method requires well-behaved, operationally stable potentiometric probes to achieve the outstanding precision that this method should provide.

## Supporting Information Available

Detailed schemes and pictures of the CapaBoard, potentiometric calibrations with the pH glass electrode, pH titrations of the buffers and current trace when performing a coulometric calibration with the pH glass electrode without the voltage follower (PDF)
