# Supplementary material for: Increasing the Sensitivity of pH Glass Electrodes with Constant Potential Coulometry at Zero Current
Source: Anal Chem. 2024 Apr 9;96(16):6436–43. doi: 10.1021/acs.analchem.4c00592 (PMC11044110; doi:10.1021/acs.analchem.4c00592)
Supplement: Supplementary file 1 — ac4c00592_si_001.pdf [file ac4c00592_si_001.pdf]

Supporting Information for

Increasing the Sensitivity of pH Glass Electrodes with Constant Potential Coulometry at Zero Current

Robin Nussbaum, Stéphane Jeanneret, Eric Bakker*

Department of Inorganic and Analytical Chemistry, University of Geneva, CH-1211 Geneva, Switzerland

*Email: Eric.Bakker@unige.ch

Table of Contents

Figure S1 .... S3
Figure S2 .... S3
Figure S3 .... S4
Figure S4 .... S5
Figure S5 .... S6
Figure S6 .... S6
Figure S7 .... S7

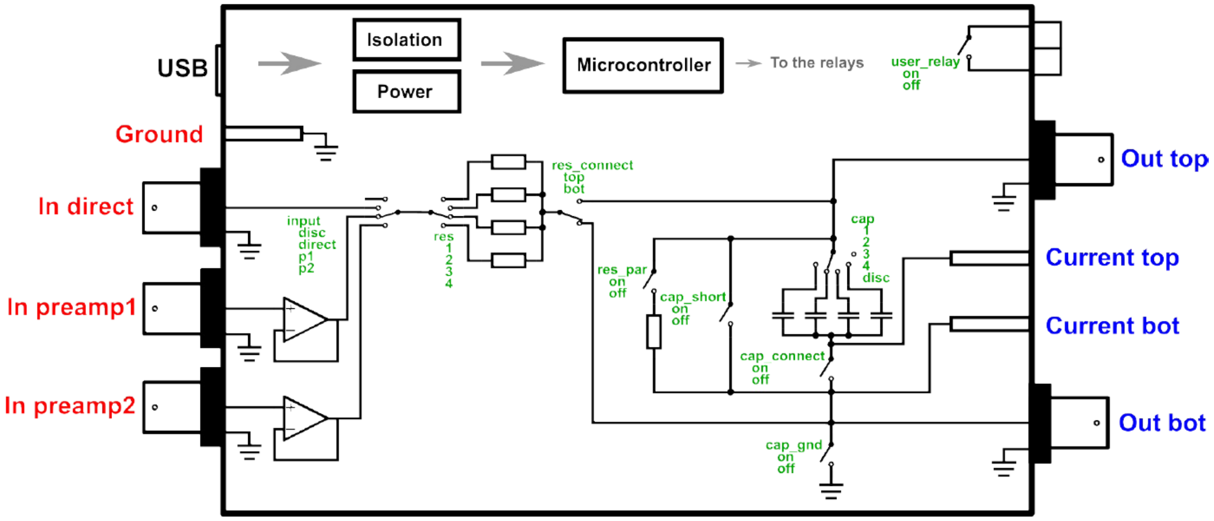

Figure S1. Scheme of the electronic circuit in the CapaBoard. In this work, the RE and CE cables of the potentiostat were connected to the *Out top* input. The WE cable was connected on the *Ground.* If not specified otherwise, the reference electrode was connected on the *Ground* while the ISE was connected to the *In preamp 1*.

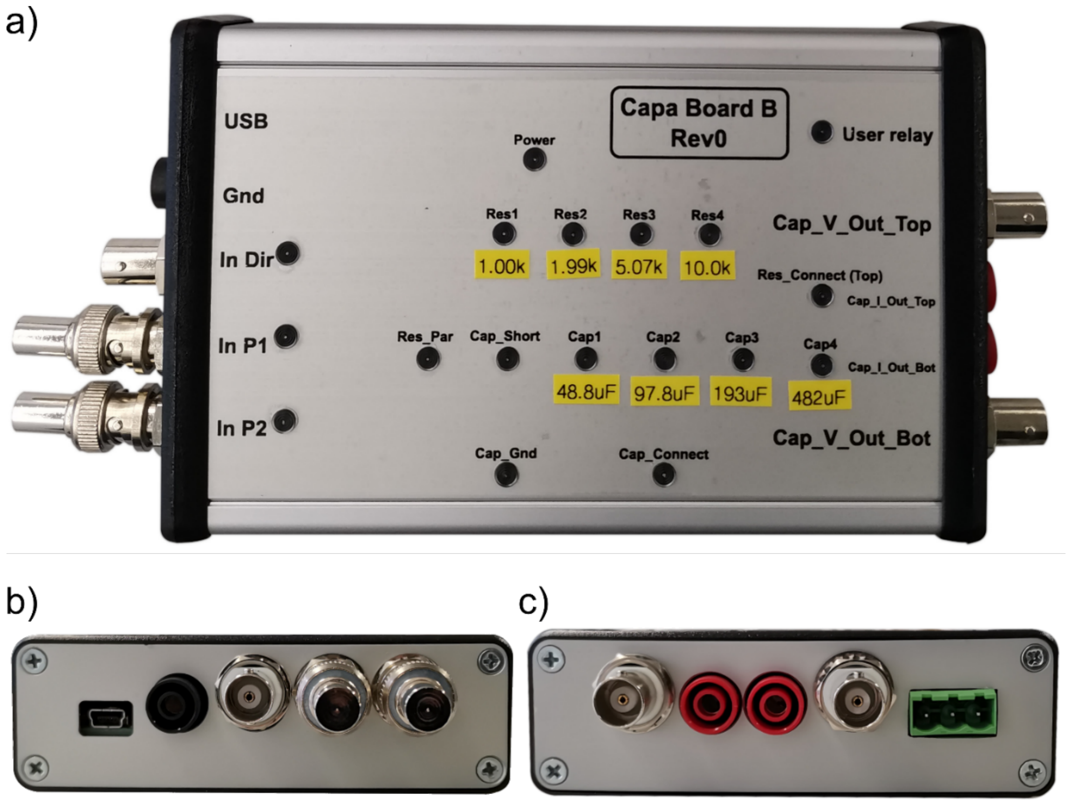

Figure S2. Pictures of the CapaBoard from a) top, b) left side and c) right side.

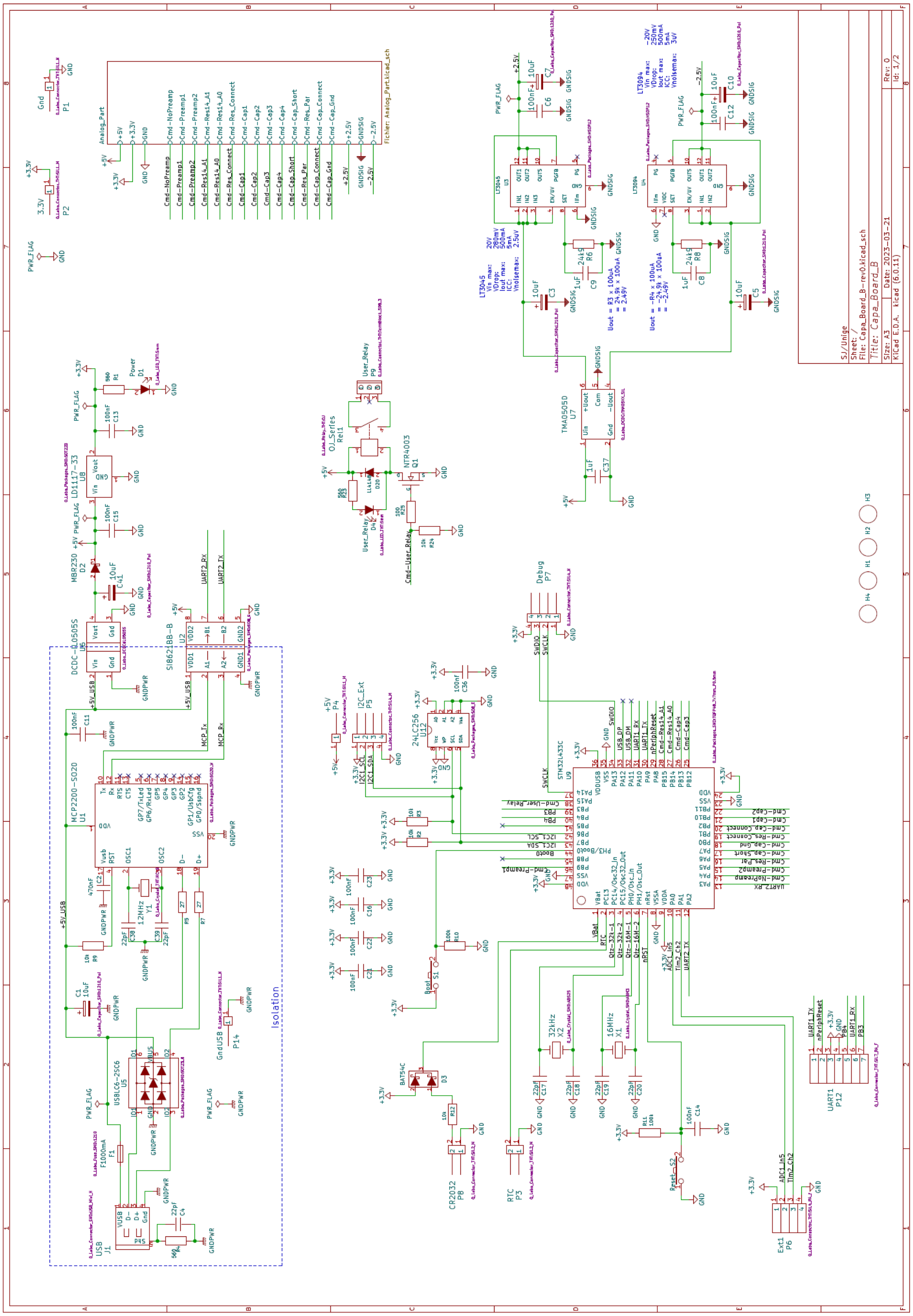

Figure S3. Detailed scheme of the CapaBoard (part 1).

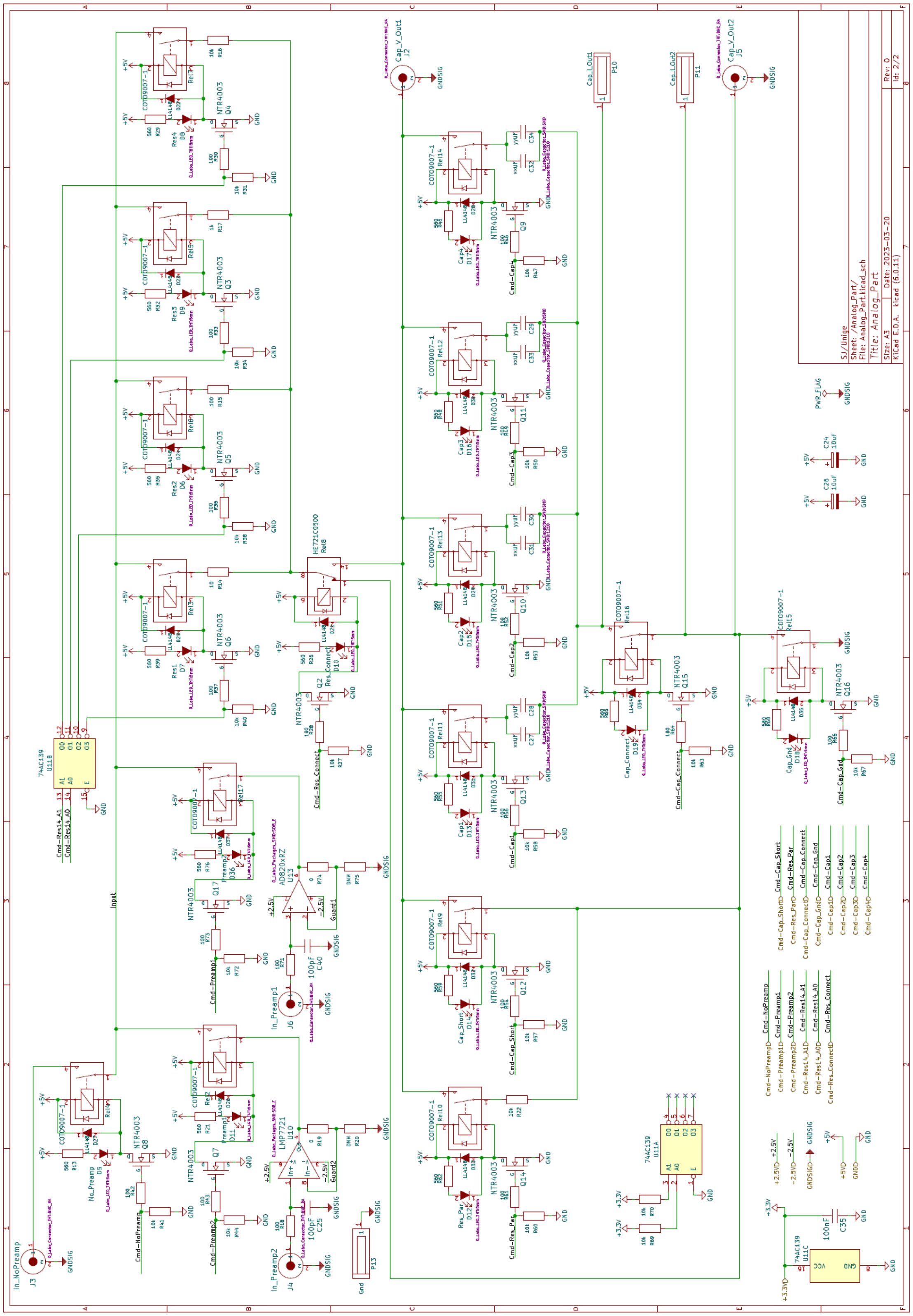

Figure S4. Detailed scheme of the CapaBoard (part 2).

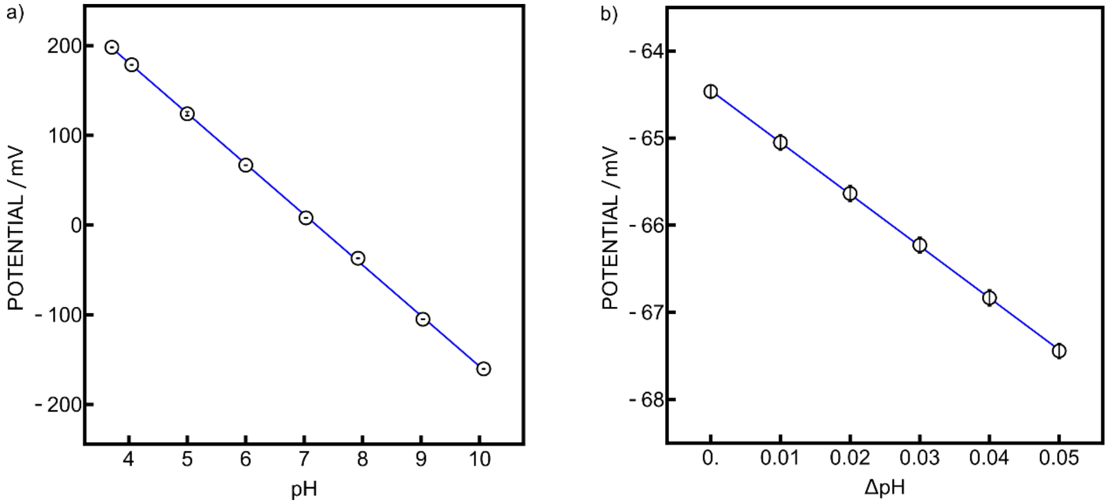

Figure S5. Potentiometric calibration using pH glass electrodes in a) 40 mM universal buffer and b) in 20mM boric acid, 10 mM NaCl, pH 8.21.

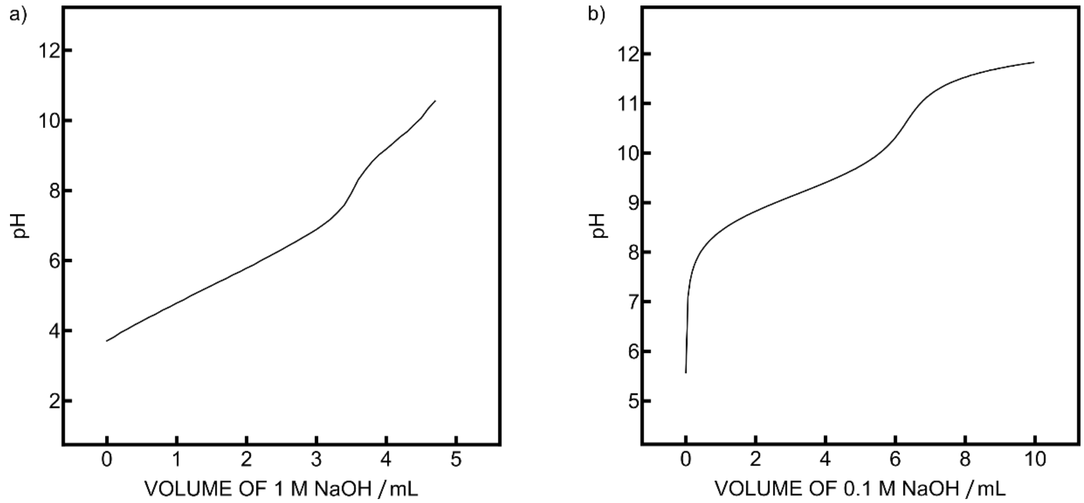

Figure S6. Automated pH titration of a) 40 mM universal buffer and b) 20 mM boric acid, 10 mM NaCl. The titrations took 40 and 10 minutes, respectively.

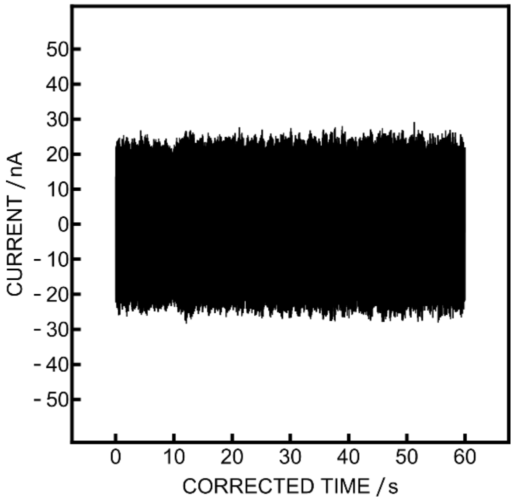

Figure S7. Current trace observed during capacitive pH calibration without the voltage follower (direct input).
